# Transgene Bioconfinement: Don’t Flow There

**DOI:** 10.3390/plants12051099

**Published:** 2023-03-01

**Authors:** Jessica N. Stockdale, Reginald J. Millwood

**Affiliations:** Department of Plant Sciences, University of Tennessee (UTK), Knoxville, TN 37996, USA

**Keywords:** bioconfinement, transgenic plants, GE crops, gene flow, male sterility, transgene excision, delayed flowering

## Abstract

The adoption of genetically engineered (GE) crops has led to economic and environmental benefits. However, there are regulatory and environmental concerns regarding the potential movement of transgenes beyond cultivation. These concerns are greater for GE crops with high outcrossing frequencies to sexually compatible wild relatives and those grown in their native region. Newer GE crops may also confer traits that enhance fitness, and introgression of these traits could negatively impact natural populations. Transgene flow could be lessened or prevented altogether through the addition of a bioconfinement system during transgenic plant production. Several bioconfinement approaches have been designed and tested and a few show promise for transgene flow prevention. However, no system has been widely adopted despite nearly three decades of GE crop cultivation. Nonetheless, it may be necessary to implement a bioconfinement system in new GE crops or in those where the potential of transgene flow is high. Here, we survey such systems that focus on male and seed sterility, transgene excision, delayed flowering, as well as the potential of CRISPR/Cas9 to reduce or eliminate transgene flow. We discuss system utility and efficacy, as well as necessary features for commercial adoption.

## 1. Introduction

In the US, more than 90% of commercially cultivated crops are genetically engineered (GE) [1]. These crops confer well-characterized transgenic traits, primarily insect and herbicide resistance, that have been engineered in a few crop species with low potential for gene flow [1]. Over the last three decades, transgene movement beyond cultivation has been a concern associated with GE crops [2,3,4]. Transgene flow may occur via the dispersal of pollen, seed, or vegetative-propagules, although pollen-mediated gene flow is commonly described as the major route of concern [4,5]. Indeed, much research has focused on potential environmental impacts of transgene movement beyond cultivation [6,7,8] as well as the potential for the establishment of free-living transgenic populations [9,10,11,12]. Transgene flow has been documented for a few crops, yet few free-living transgenic populations have been identified and there have been no reports of measurable negative environmental impacts [13,14,15,16,17,18]. This may be due to the reproductive biology of current GE crops and the area of cultivation. For example, many of these crops are highly domesticated, primarily self-pollinate, and are cultivated in regions with few to no sexually compatible wild relatives [19].

Gene flow is more likely to occur in crop species with high outcrossing frequencies often associated with wind-pollinated crops and obligate outcrossing species, such as switchgrass (*Panicum virgatum* L.). The probability of interspecific hybridization is also higher if there are wild relatives native to the region of cultivation [20]. Additionally, certain transgenes and their conferred traits may also raise biosafety concerns in regard to environmental and ecological risk if they were to escape to unmanaged wild relative populations [19]. For example, certain transgenes may provide advantageous traits, such as improved tolerance to abiotic stress conditions including drought, salinity, or low and high temperatures [20,21]. There is concern that GE trait introgression into wild populations could provide a competitive advantage to transgenic hybrids under stress conditions. This may lead to potential ecological consequences such as decreased biodiversity or possibly local population extinction [22,23].

To address these concerns several transgene confinement approaches have been proposed. The implementation of physical or biological confinement (bioconfinement) strategies could prevent transgene flow. Physical confinement approaches utilize physical barriers such as enclosures, fencing, flower removal, and geographic isolation that provide spatial separation from sexually compatible crops and wild relatives. These approaches are often not practical solutions for large-scale commercialization in terms of physical labor and facility requirements [24]. Alternatively, biological confinement approaches offer feasible and sustainable strategies to contain transgenes. Since the early 1990s, bioconfinement approaches have been studied for their potential to limit or eliminate transgene flow. To date, none are commonly used in commercial crops. The adoption of a bioconfinement system would allow new GE crops to reach the market that may not otherwise. Numerous technologies have been proposed through various approaches including maternal inheritance, genome incompatibility [25], apomixis [26], cleistogamy [27,28], and total sterility, but their application is limited. In this review, we survey efficient and widely applicable bioconfinement technologies such as male and seed sterility, transgene excision, and delayed flowering. These approaches have been achieved through genetic engineering, including type II clustered regularly interspaced short palindromic repeat/CRISPR-associated protein 9 (CRISPR/Cas9) gene editing. We also discuss the most effective bioconfinement methods developed to date and compare their potential to eliminate transgene flow. Lastly, perspectives are provided for features we believe necessary for commercial adoption of a bioconfinement system.

## 2. Engineered Reproductive Sterility

### 2.1. Male Sterility

Engineered male sterility is a bioconfinement approach that can be designed to yield pollenless flowers or flowers with sterile transgenic pollen. Generally, GE male sterile phenotypes can be produced through the tightly-controlled and targeted expression of genes that yield products cytotoxic to reproductive tissues [21,25,29]. To date, genes used for engineered male sterility include *barnase*, *Diphtheria toxin A-chain*, and the endonuclease *Eco*RI. Each have been expressed in several plant species with varying results which are described below.

#### 2.1.1. Barnase-Induced Male Sterility

**Figure 1:**
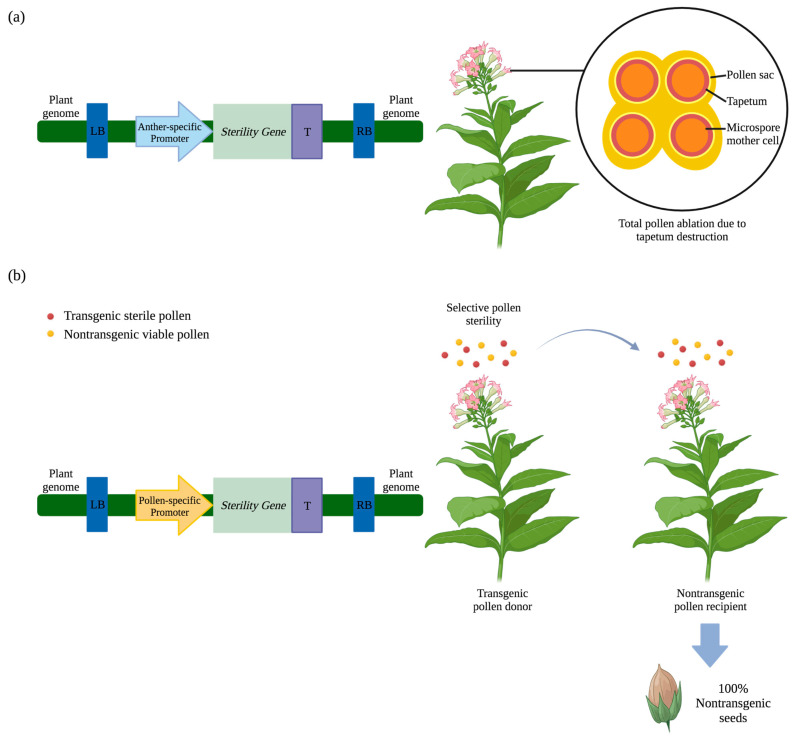
Engineered male-sterility approaches. (**a**) A sterility gene, such as *Barnase*, is located between an anther-specific promoter and a terminator (T) within the left (LB) and right borders (RB) on T-DNA inserted into the plant genome. *Barnase* expression prevents pollen formation by degrading RNA in the tapetal cells, the nutritive cells surrounding microspore mother cells. (**b**) A sterility gene, such as *Eco*RI endonuclease, is located between a pollen-specific promoter and a terminator within the left and right borders on the T-DNA, and in this example is inserted once in the plant genome. In this theoretical scenario, *Eco*RI expression leads to the death of transgenic pollen grains and nontransgenic pollen remains viable. Pollen movement from transgenic to nontransgenic plants yields 100% nontransgenic seeds. Figure created with Biorender.com, accessed on 22 February 2023.

The bacterial gene *barnase* (from *Bacillus amyloliquefaciens*) has been used to generate male sterile plants. Barnase is a ribonuclease that, when expressed in plant cells, leads to RNA degradation and subsequent cell death [30]. When utilized for engineered male sterility, barnase expression has been targeted to tapetal cells which form the nutritive cell layer surrounding microspore mother cells [31,32]. These cells are necessary for viable pollen development [31]. When barnase is expressed in this manner, tapetal cell death prevents microspore development and confers total pollen ablation (Figure 1a) [30]. Fertility can be restored by crossing with restoration lines genetically engineered to express barstar (also from *Bacillus amyloliquefaciens*) which acts as a repressor of barnase [33,34].

Experimental results in tobacco (*Nicotiana tabacum* L.) and canola (*Brassica napus* L.) under the control of the tapetum-specific promoter TA29 (derived from tobacco) revealed barnase expression conferred completely pollenless plants in 92% and 77% of transgenic events, respectively [30]. Except for the absence of pollen, these transgenic plants were indistinguishable from nontransgenic tobacco controls in vegetative and floral morphology and displayed a similar growth rate and height through all developmental phases.

Using this approach, barnase has been used for male sterile plant production in several monocot species including wheat (*Triticum aestivum* L.) [35], creeping bentgrass (*Agrostis stolonifera* L.) [36], and rice (*Oryza sativa* L.) [37]. In wheat, tapetum-specific promoters ca55 (derived from maize (*Zea mays* L.)), and T72 and E1 (derived from rice) were used to drive barnase expression [35]. Out of 29 transgenic events, 11 were chosen for phenotypic analysis and crossing experiments. Two events contained barnase under the control of the promoter E1, six were under the control of the T72 promoter, and the remaining three events utilized the ca55 promoter. All transgenic events displayed a completely pollenless phenotype that was stably inherited across two generations of backcrossing except for one event (T72) which produced fertile transgenic progeny in cross-pollination experiments. These transgenic plants displayed normal phenotypes and were comparable to wild-type plants in height, leaf size, and tiller count. When expressed in creeping bentgrass using the tapetum-specific promoter TAP (derived from rice), barnase expression led to significantly reduced pollen production in 90% of transgenic lines [36]. For plants that produced pollen, viability was measured and all pollen grains tested were non-viable. No off-target expression was reported, and the male sterile phenotype was stably inherited in progeny when backcrossed to nontransgenic creeping bentgrass. When barnase was expressed in rice using the pollen-specific promoter Zm13 (derived from maize) [37], transgenic events were crossed to nontransgenic rice. The resulting progeny produced 50% viable nontransgenic pollen. Transgenic pollen expressing barnase was nonviable which is expected for transformants with a single T-DNA copy. The cross-pollination experiments also revealed that pollen-specific expression of barnase had no effect on seed yield, and plants displayed normal growth and development.

Barnase has also been expressed in male-specific tissues of GE trees such as pine (*Pinus radiata* D. Don) [38], eucalyptus (*Eucalyptus globulus* Labill.) [38], and hybrid poplar (*Populus tremula* × *Populus tremuloides*) [39]. In pine and eucalyptus, a male cone-specific promoter PrMC2 (derived from pine) was used to express the modified barnase coding sequence barnaseH102E [38]. BarnaseH10E2 contained a single amino acid substitution which was expected to reduce RNAse activity and prevent off-target expression. When expressed in pine, 97% of transgenic events did not produce pollen. Nine of these transgenic events were studied under field conditions, where pollenless cones were observed over a 2-year study. Similar results were observed in eucalyptus where 95% of transgenic events analyzed were pollenless. Under field conditions, 16 of 17 transgenic events produced pollenless cones over a four-year study. Cone dissection revealed that one transgenic event produced pollen, but the pollen was non-viable. Additionally, vegetative growth and development were normal for both transgenic pine and eucalyptus. Barnase was also expressed in poplar hybrids (*Populus tremula* × *Populus tremuloides*) under the control of the tapetum-specific promoter TA29 [39]. Throughout a four-year field study, two of six transgenic trees were completely pollenless and four transgenic trees released approximately six pollen grains per catkin (a flower cluster found in certain tree species) during the first two years of the study. Transgenic pollen production was significantly less compared to nontransgenic trees which produced approximately 73,000 to 85,000 pollen grains per catkin during the same period. In the final two years of the study, no pollen was produced in transgenic trees whereas 775,000 to 2,700,000 pollen grains per catkin were produced in nontransgenic controls. Unfortunately, plant growth was negatively affected as determined by stem volume (height × diameter^2^) measurements. Of 18 transgenic poplar events, 17 were significantly smaller compared to nontransgenic controls. Because growth of transgenic poplar expressing the β-glucuronidase (GUS) reporter gene was similar to that of nontransgenic controls, this phenotype was attributed to off-target barnase expression and possibly worsened by field conditions.

In summary, the targeted expression of barnase yields pollenless flowers or sterile pollen production and may be an effective strategy to prevent transgene flow with little to no negative effects on plant growth and development. Although examples of off-target expression were observed in few transgenic events, the use of barnase for bioconfinement is applicable across many crop species, stable across several generations, and can effectively confer male sterility. Nonetheless, this bioconfinement system can be further optimized through the use of synthetic promoters that provide increased sterility gene expression to targeted tissues for enhanced efficacy in a wide array of plant species.

#### 2.1.2. Diphtheria Toxin A-Chain-Induced Male Sterility

Another well-characterized cytotoxic gene used for engineered male sterility is *Diphtheria toxin A-chain* (*DTA*) (from *Corynebacterium diphtheriae*). When expressed in cells, DTA catalyzes ADP-ribosylation and inactivation of elongation factor 2 [40]. This action inhibits protein synthesis resulting in cell death. Targeted *DTA* expression has been used for cell–cell interaction and functional analysis studies [41,42]. The characterization of tapetum- and pollen-specific promoters through the expression of DTA in transgenic plants was found to yield pollenless and pollen-sterile plants with minimal to no effects on plant growth [41,42]. For this reason, targeted DTA expression has been adopted as a bioconfinement strategy through male sterile plant production. Male fertility can be restored by crossing with transgenic lines engineered to express a diphtheria toxin repressor (DTxR) (also from *Corynebacterium diphtheriae*) [43].

Tapetum-specific expression of DTA by the promoter TA29 was analyzed in seven transgenic tobacco plants, all of which contained a single T-DNA copy [42]. From these transformants, anther development was identical to that of nontransgenic control plants except for the destruction of the tapetum and collapse of the pollen sac resulting in pollenless plants. No off-target expression or negative effects on growth were reported suggesting TA29 promoter activity is localized to tapetum cells.

Another study used the pollen-specific promoter Lat52 derived from tomato (*Solanum lycopersicum* L.) to drive the expression of DTA in 20 transgenic tobacco events [41]. Of the 20 events, 16 were male-sterile and displayed one of two phenotypes. Half of the events displayed a pollenless phenotype, whereas the other half had a significant reduction in pollen grains of which 50% were nonviable. Two transgenic events that produced 46.3% and 50.9% nonviable pollen were selected for further analyses, and the pollen-sterile phenotype was stably inherited through four generations of self-pollination. Additionally, two of the 20 transgenic events displayed off-target expression which resulted in decreased flower size.

Both tapetum- or pollen-DTA expressing plants are phenotypically similar to barnase expressing male-sterile plants. Tapetum- and pollen-specific expression of DTA yielded male-sterile tobacco with few to no off-target affects [41,42]. However, concerns may arise in regard to the use of DTA in crops. Although a single molecule of DTA can induce cell death, diphtheria toxin B chain is required to transport DTA into cells [40,44]. The use of DTA in this manner may raise concerns regarding potential pollen-feeding insects and human health effects even though DTA is not highly toxic alone. Despite its potential, these concerns may create roadblocks when used as a bioconfinement strategy.

#### 2.1.3. *Eco*RI Endonuclease-Induced Male Sterility

Another gene studied for bioconfinement purposes is the *Eco*RI endonuclease (from *Escherichia coli*) which has been expressed in pollen to confer selective male sterility in tobacco (Figure 1b) [45]. *Eco*RI endonuclease is a well-characterized type II restriction endonuclease that creates a double-stranded break at the 5′-GAATTC-3′ recognition sequence [46]. The expression of *Eco*RI in pollen causes repeated double-stranded breaks in the pollen nuclear genome which induces cell death [45].

Pollen-specific expression of *Eco*RI endonuclease by the Lat52 promoter conferred selective male sterility in tobacco, and all transformants displayed over 99% and up to 100% bioconfinement efficacy when used as the pollen donor source and crossed to nontransgenic tobacco plants under greenhouse and field conditions [45]. Additionally, bioconfinement efficacy was stable across generations and transgenic plants were indistinguishable from nontransgenic plants in regard to growth and vegetative morphology. Thus far, this approach has been characterized in tobacco, but more research is needed in other species since recognition-site frequency of restriction endonucleases and promoter activity can vary. Furthermore, this approach is advantageous for pollen-feeding insects since it permits pollen production, whereas other male-sterility approaches may be food-source limiting.

### 2.2. Conditional Seed Sterility

Conditional seed sterility is another promising bioconfinement approach that was originally designed to protect intellectual property in genetically engineered plants [47,48,49]. Such approaches are referred to as genetic use restriction technologies (GURTs). GURTs are often designed as an inducible system that activates sterility gene expression under specific conditions [19,50]. One of the earliest GURTs, termed Genesafe technology, was originally developed in the 1990’s to prevent the unauthorized use of transgenic seeds by inducing seed sterility in the next generation [47,48,49,51]. Although promising, Genesafe was never commercialized. This was partially due to public opposition since growers could not save and reuse seeds [19,50,52]. However, this approach holds great potential as a bioconfinement system specifically for vegetatively or clonally propagated crops such as switchgrass, certain hemp cultivars (*Cannabis sativa* L.), and poplar.

**Figure 2:**
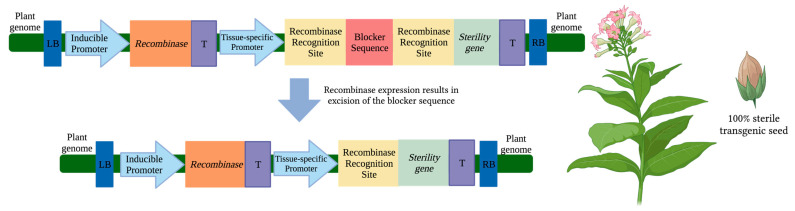
Conditional sterility via inducible system activation of sterility genes. Within the left (LB) and right borders (RB) of the T-DNA located in the plant genome, an inducible promoter is located upstream of a recombinase gene and terminator (T). An external stimulus is applied to induce recombinase gene expression. The recombinase enzyme subsequently excises the blocker sequence by cutting DNA segments at flanking recognition sites. The tissue-specific promoter (ex. seed-specific) is moved into close proximity of the sterility gene and transcription can occur. The sterility gene product leads to cell death. In this theoretical example, a homozygous tobacco line with a seed-specific promoter driving sterility gene expression leads to 100% sterile transgenic seeds. Figure created with Biorender.com, accessed on 18 January 2023.

GURT designs often require activation by an external stimulus, such as a chemical or temperature treatment, which would activate an engineered genetic circuit and inhibit seed germination in the subsequent generation (Figure 2). These GURTs are comprised of four components: (i) a genetic switch which is inducible and can activate or repress the trait switch; (ii) a trait switch which is typically a recombinase gene responsible for removing a blocker sequence; (iii) a blocker sequence flanked by recombinase recognition sites (located between the seed-specific promoter and sterility gene); and (iv) a sterility gene controlled by a seed-specific promoter [50,53]. Altogether, the application of a stimulus activates the genetic switch, which amplifies the trait switch and removes a blocker sequence to bring the seed-specific promoter and sterility gene in close proximity [50,52]. Seed-specific expression of the sterility gene renders transgenic seeds sterile.

The original Genesafe prototype was a repressible system and required hybridization of two parent plants (P1 and P2) to produce inactive, inducible seeds carrying the entire Genesafe system [47,53]. P1 carried the seed-specific promoter and sterility gene divided by the blocker sequence that produces a repressor protein. P2 carried a recombinase gene that, without application of a stimulus, was repressible by the blocker sequence in P1 when hybridized. After hybridization, a stimulus treatment would release the repressor, and the recombinase would excise the blocker sequence. As a result, the sterility gene would be expressed in the next generation of seeds. This design was tested in tobacco using late embryo-specific promoters LEA4A and LEA14 derived from cotton (*Gossypium herbaceum* L.) to drive expression of the sterility gene *saporin* (from *Saponaria officinalis*), a ribosome-inactivating protein that inhibits protein synthesis when expressed in plant cells [54]. The blocker sequence was successfully excised using the recombinase system Cre/*loxP*; however, seed-sterility was not observed [55]. This was attributed to the inefficient activity of the cotton-derived promoter in tobacco, as well as insufficient saporin production. This design was not further tested because a new and simpler design was created which used a single parent to carry the Genesafe system. The new design was chemically inducible, where application of a stimulus would permit expression of the recombinase gene and excise the blocker sequence [47,53]. As a result, the seed-specific promoter would express the sterility gene and was expected to produce non-germinable seeds in the following generation. This design can be altered to use other components to make up the chemically-inducible system but was described with the Cre/*loxP* recombinase system, LEA promoters, and the *saporin* gene. However, no data were provided for seed sterility.

Although Genesafe was not previously commercialized for its original purpose, it could be immensely useful for bioconfinement if used in clonally or vegetatively propagated crops. Improved tools and system components are available that can be used to optimize the system. Specifically, efficient recombinase systems can be employed such as gene-deletor technology (described in Section 3) which has displayed up to 100% transgene excision from pollen and/or seeds [56]. Improved inducible- and tissue-specific synthetic promoters are available which have been optimized for strong inducible and highly specific spaciotemporal expression [57,58,59,60]. With optimized components and new designs, Genesafe holds promise as a bioconfinement system that can be incorporated into a wide array of crops.

## 3. Transgene Excision

**Figure 3:**
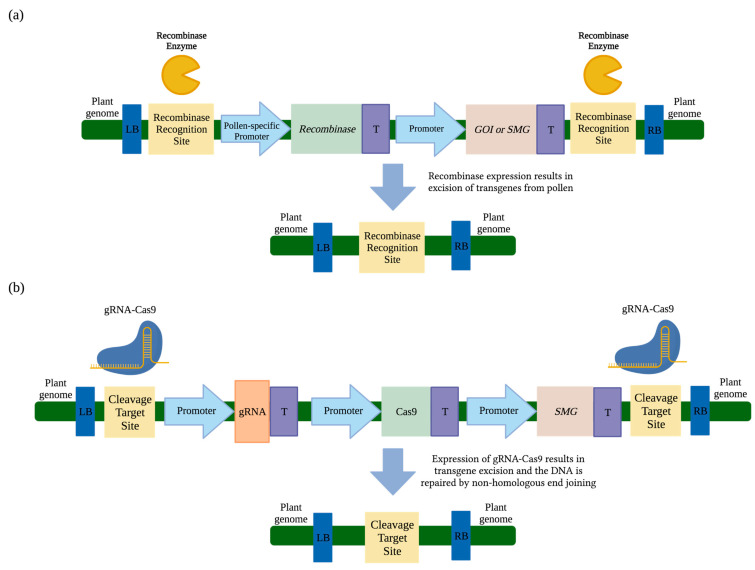
Transgene excision by site-specific recombinase and CRISPR/Cas9 gene editing. (**a**) Recombinase expression is controlled by a pollen-specific promoter. In pollen, the recombinase recognizes sites flanking the gene of interest (GOI) or selectable marker gene (SMG) and the recombinase expression cassettes and removes the DNA between the recognition sites. One recognition site and left (LB) and right (RB) borders remains while the DNA removed is degraded within the plant cell. (**b**) CRISPR/Cas9 excision utilize cleavage target sites (CTSs) that flank the guideRNA (gRNA), Cas9 enzyme, and SMG expression cassettes. The CTSs are comprised of identical DNA sequences. Expression of a single gRNA and Cas9 enzyme excises the T-DNA between the CTSs. In this example, cleaved DNA ends are repaired by non-homologous end joining, leaving behind one CTS and left and right borders and the DNA removed is degraded within the plant cell. Figure created with Biorender.com, accessed on 22 February 2023.

Transgene excision has been proposed as a strategy to eliminate transgene flow. This strategy utilizes inducible- or tissue-specific promoters to express a site-specific recombinase. The DNA targeted for excision is flanked by site-specific recombinase recognition sites during design and cloning of the transformation construct. Depending on the placement of the recognition sites, this strategy can be used to remove transgenes such as selectable marker genes (SMGs) or entire T-DNA cassettes. The excised DNA is degraded by non-specific nucleases within the cell, and components that were not located between the recognition sites would remain in the genome including the left and right T-DNA borders and one recombinase recognition site (Figure 3a) [61,62].

### 3.1. Transgene Excision via Cre/loxP Recombinase

The most studied and best characterized bidirectional recombinase systems include Cre-*loxP* (from bacteriophage P1), FLP-*FRT* (from *Saccharomyces cerevisiae*), and R-*RS* (from *Zygosaccharomyces rouxii*) [63,64,65]. Tyrosine recombinases that recognize relatively short sequences (34–58 base pairs) are used in these systems to mediate DNA cleavage [63,64,65]. Dependent on the orientation of the recognition sites relative to each other, integration, excision, or inversion of targeted sequences of DNA can occur [66]. Although all of these outcomes have been observed, excision and degradation of the targeted DNA is observed most often since it is kinetically favored [66].

The Cre-*loxP* recombinase system has been studied in Arabidopsis (*Arabidopsis thaliana* L.) [67] and tobacco [67,68,69,70] studies, where pollen was targeted for transgene excision to prevent pollen-mediated gene flow [67,70]. In Arabidopsis and tobacco greenhouse experiments, the microspore-specific promoter NTM19 (derived from tobacco) was used to drive Cre expression and produce transgene-free pollen [67]. Transgenic Arabidopsis and tobacco plants were used in self- and cross-pollination experiments, followed by backcross experiments. Backcross experiments displayed 100% transgene excision in three Arabidopsis lines. In transgenic tobacco, over 99% transgene excision was observed in five lines. In addition to high transgene excision efficiency, plants displayed normal growth and development. Another study used the Cre/*loxP* recombinase system to excise the SMG from transgenic tobacco through the use of the pollen- and embryo-specific promoter DLL (derived from Arabidopsis) to drive Cre expression [70]. Eight transgenic tobacco lines were self-pollinated and 99% of the progeny displayed partial or complete excision of the SMG. However, there was notable variation across transgenic lines, ranging from 3.8% to 81.5% excision efficiency. Four of these lines were used in cross-pollination experiments as pollen donors to nontransgenic tobacco, where complete or partial excision of the SMG was observed in 99% of plants. These experiments also displayed variation across transgenic lines which ranged from 40% up to 96.2% excision efficiency. High excision efficiency has been observed and supports the potential of the recombinase system for transgene excision; however, the variation among lines should be addressed by utilizing promoters that provide consistent and targeted high expression levels.

Seed-specific expression of the Cre/*loxP* recombinase system was also studied to prevent seed-mediated transgene flow. In tobacco (*Nicotiana benthamiana* L.), the seed-specific promoter napin (derived from canola) was used to drive Cre expression and determine the efficiency of SMG excision from seeds [69]. Tobacco was co-transformed with two plasmids, the first of which contained the SMG flanked by the *loxP* recognition sites and the second construct contained Cre driven by the napin promoter. A total of 10 lines were co-transformed, self-pollinated, and the progeny were subjected to analysis. Excision of the SMG occurred in all 10 lines, eight of which displayed 100% excision efficiency and two lines displayed 76.8% and 93.1% excision efficiency. Additionally, all transgenic lines were normal in regard to vegetative and reproductive morphology. The expression of the Cre/*loxP* recombinase system was also studied in tobacco (*Nicotiana tabacum* L.) under the control of the seed-specific promoter BcNA1 (derived from canola) to remove the SMG [68]. Eight transgenic lines were obtained and self-pollinated, and progeny were subjected to a germination assay on selection media to determine whether excision had occurred. In four of the transgenic lines, 100% excision was observed. In the remaining transgenic lines, excision occurred at a rate of 25.4%, 29.9%, 59.6%, and 71.8%. Additionally, all transgenic lines appeared normal in regard to plant growth and fertility. In transgenic canola, Cre expression was driven by the embryo-specific promoter USP88 (derived from *Vicia faba* L.) to excise the SMG and produce SMG-free progeny [71]. Progeny from eight transgenic lines were analyzed and an average of 32.7% excision efficiency was observed, none of which were reported to have adverse morphological features. Although these lines were able to produce SMG-free progeny, the average excision rates were relatively low compared to other studies. To improve this system, stronger and more tightly-controlled promoters should be selected to drive Cre expression.

Both pollen- and seed-specific expression of the Cre/*loxP* recombinase system have displayed up to 100% transgene excision in multiple transgenic lines. However, significantly lower efficiency has been observed in several lines. To reduce variability among lines, it is apparent that design changes, specifically for promoters that drive recombinase expression, as well as further testing and optimization are needed to ensure high and consistent levels of transgene excision in all transgenic lines. If consistency is achieved, the Cre/*loxP* recombinase system may be a useful bioconfinement tool.

### 3.2. Transgene Excision from Pollen via CinH-RS2

Other well-characterized recombinase systems are CinH-*RS2* (from *Acinetobacter* plasmids pKLH2 and pKLH204) and ParA-*MRS* (from the broad host range plasmid RK2), which are unidirectional recombinase systems and use serine recombinases to irreversibly excise targeted sequences of DNA [72,73,74]. Recombinases of the CinH-*RS2* and ParA-*MRS* recombinase systems recognize 113 and 133 base pair sequences, respectively, which are much longer than the tyrosine-derived recombinase systems previously described and are expected to decrease potential off-target DNA cleavage [75,76].

Transgene excision from pollen has been studied in tobacco using the CinH-*RS2* recombinase system [61]. In transgenic tobacco, the pollen specific Lat52 promoter (derived from tomato) was used to drive expression of CinH recombinase. The *RS2* recognition sites flanked all transgenes in the construct and were within the T-DNA borders. A green fluorescent protein (GFP) visual marker gene was located in this region and expression was driven by the Lat59 pollen-specific promoter (derived from tomato). In this design, successful excision would prevent expression of GFP in pollen. Pollen grains from five transgenic lines with a single T-DNA copy were collected and analyzed, three of which displayed less than 1% GFP-expressing pollen and the other two lines displayed 1.94% and 17.48% GFP-expressing pollen. Furthermore, transgenic plants were phenotypically indistinguishable from nontransgenic controls in regard to plant growth and reproduction. Thus, the authors concluded that highly efficient transgene excision is possible, and this approach holds promise for transgene bioconfinement.

Based on these results, the CinH-*RS2* recombinase system can effectively excise transgenes from pollen without negatively impacting plant growth. The pollen analysis showed promising results. However, cross-pollination experiments should be performed to confirm that transgenes are not present in the progeny. Further research is also needed to evaluate design and system components for improvements to achieve a 100% excision efficiency as observed in other designs.

### 3.3. Transgene Excision from Pollen and/or Seeds via Fused loxP-FRT Recombinase System

Gene-deletor technology is a term used to describe a site-specific recombinase system that uses fused *loxP*-*FRT* recognition sites on either side of the transgene (s), along with expression of either Cre or FLP recombinase genes [56]. Cre and/or FLP recombinases were driven by pollen-specific promoters Lat52 (derived from tomato) or BGP1 (derived from *Brassica campestris*), or pollen- and seed-specific promoter PAB5 (derived from Arabidopsis) to remove transgenes from pollen and/or seeds of GE tobacco. Gene-deletor technology displayed 100% transgene excision in pollen and seeds from multiple transgenic tobacco events. Specifically, analysis of progeny produced from self- and cross-pollination experiments revealed that constructs with fused *loxP*-*FRT* recognition sequences and promoter:recombinase combinations BGP1:FLP, PAB5:FLP, and Lat52:Cre displayed 100% excision efficiency in multiple lines. Additionally, no deleterious effects on vegetative or reproductive growth were reported. In the same study, the promoter:recombinase combination BGP1:FLP was used with only the *FRT* recognition site and displayed 99% excision efficiency, a slightly lower rate than when both *loxP*-*FRT* recognition sites were present (100%). The study also examined how the simultaneous expression of Cre and FLP recombinases driven by Lat52 affected transgene excision in the presence of fused *loxP*-*FRT* recognition sites. This design produced up to 45% transgene excision, a significantly lower rate from Cre expression alone.

These studies suggest gene-deletor technology increased excision efficiency and can effectively eliminate transgene flow in tobacco without negatively impacting plant growth and development. The promoter:recombinase combinations with high performance should be tested in other crop species to determine if efficacy is comparable.

### 3.4. CRISPR/Cas9-Mediated Transgene Excision

In recent years, type II clustered regularly interspaced short palindromic repeat/ CRISPR-associated protein 9 (CRISPR/Cas9) has been utilized for transgene excision. It requires two Cas9 cleavage target sites (CTS) on either side of the target DNA and a guide RNA (gRNA)-Cas9 assembly that recognizes and cleaves the CTSs. The excised DNA is degraded by non-specific nucleases in the cell. Plasmid construct components that were not located between the CTSs would remain in the genome (Figure 3b) [77].

In one study, CRISPR/Cas9 transgene excision efficacy was examined for the excision of SMGs in transgenic rice [78]. To do so, callus of transgenic rice that constitutively expressed GUS was transformed with a CRISPR/Cas9 construct. The cleavage target sites were located at the ends of the 1.6 kb *GUS* gene. Since the *GUS* gene is expected to be removed from the genome, successful excision would be determined by lack of GUS expression. In this experiment, a total of 34 plants were produced from 12 transgenic events. Three of these events displayed 100% transgene excision. Despite high excision efficiency, the authors report that normal vegetative growth was observed in four of 34 transgenic plants and only two plants produced a small quantity of seeds. No specific details were provided for the adverse phenotypes and off-target Cas9 activity. While this study displayed 100% transgene excision from multiple transgenic events, reports of no phenotypic and off-target effects are necessary to eliminate concerns of potential negative outcomes.

Another study also aimed to excise SMGs from rice through CRISPR/Cas9-mediated transgene excision [79]. The promoter Pssi (derived from rice) was expected to express strongly in stem, shoot tip, and inflorescence tissue, and was therefore selected to drive Cas9 nuclease expression. Transgenic rice was generated to constitutively express GUS; however, the *GUS* gene was separated into two segments denoted as ‘GU’ and ‘US’, interrupted by a CRISPR/Cas9 cassette and a selectable marker gene that acted as a blocker sequence flanked by CTSs. Additionally, both ‘GU’ and ‘US’ carried a 1027 bp ‘U’ sequence as a homologous region, inducing CRISPR/Cas9-mediated homology-directed repair following excision of the blocker sequence resulting in a functional *GUS* reporter gene if excision was successful. Transgene excision occurred in 11 of 15 transgenic lines. In lines where excision was observed, T_1_ lines were produced and three were randomly selected for further analysis. In one line, 6.7% homozygous excision and 66.7% heterozygous excision was observed. Another displayed 73.3% homozygous excision and 13.3% heterozygous excision, and the last line displayed 86.7% homozygous excision. In contrast to the study previously described, no adverse effects were observed in regard to plant growth and reproduction.

CRISPR/Cas9-mediated transgene excision displayed high excision efficiency in rice. However, there are limited studies that have used CRISPR/Cas9 for transgene excision, one of which resulted detrimental effects on plant morphology and reproduction. The use of tissue-specific promoters to drive Cas9 expression, such as those that target pollen or seeds, may help reduce potential off-target effects in vegetative tissues. We believe CRISPR/Cas9-mediated transgene excision can be a useful bioconfinement system, but further optimization is needed.

## 4. Delayed Flowering

The transition from vegetative to flowering phase is a complex regulatory system controlled by microRNAs (miRNAs), transcription factor (TF) genes, and floral identity genes. miRNAs are a class of small regulatory RNAs that regulate gene expression and RNA silencing through RNA degradation or translational inhibition [80,81]. The overexpression of certain miRNAs can silence floral identity genes that may result in shifts in flowering time or reduced flower production. This approach has the potential to shift flowering time to prevent complete or partial overlap with the flowering time of sexually compatible plants, thus preventing or limiting transgene flow [82,83].

### Overexpression of miR156 Delayed Flowering

miRNA 156 (miR156) is one of several miRNAs responsible for regulating the floral transition in the shoot apical meristem [84]. miR156 expression is regulated by the age-dependent pathway, and expression is high in seedlings and declines as the plant matures. It has been characterized to repress transcripts of the *SQUAMOSA PROMOTER BINDING PROTEIN-LIKE* (*SPL*) TF family that upregulate floral identity genes *SUPPRESSOR OF OVEREXPRESSION OF CONSTANS1* (*SOC1*) and *LEAFY* (*LFY*) [84]. Thus, the overexpression of miR156 is expected to prevent the upregulation of *SOC1* and *LFY* as the plant matures, which consequently inhibits/delays the transition from the vegetative to flowering phase. For this reason, the overexpression of miR156 has been studied in switchgrass under greenhouse and field conditions to determine its potential for transgene bioconfinement via delayed flowering [85,86].

In a greenhouse study, switchgrass plants engineered to overexpress miR156 (from rice) were categorized as low, moderate, and high based on transgene expression levels [85]. Transgenic switchgrass with low expression of miR156 flowered at the same time and displayed normal reproductive growth compared to nontransgenic controls. These plants also displayed an increase in tiller number and vegetative biomass. Plants considered to have moderate expression of miR156 did not flower after 360 days and were also observed to have an increase in tiller number and vegetative biomass. However, these lines had several notable defects when compared to nontransgenic controls, including reduced plant height, decreased internode diameter, leaf blade width, and leaf sheath length. Similar to the plants categorized as moderate expression, transgenic switchgrass with high expression of miR156 did not flower after 360 days. Additionally, high expression of miR156 led to an increase in tiller number and severe defects including a reduction in plant height and biomass, as well as significantly decreased internode diameter and length, leaf sheath length, and leaf blade length and width compared to nontransgenic switchgrass [85].

Subsequently, a two-year field study was conducted using low and moderate miR156 overexpression lines to determine flowering time and biomass production under field conditions [86]. Compared to nontransgenic controls, low overexpression lines were highly variable in flowering time during the first year of establishment post-transplanting. Once these lines were established, there was no delay in flowering during the second year. Reduced biomass was observed in some plants, and fewer flowers and seeds were observed in all low expression lines. However, when flowering was monitored for moderate overexpression lines, many plants did not flower in either year of the field study. Of the plants that did flower, timing was delayed by 12 weeks in the first year and two weeks in the second year. Also, some plants displayed reduced biomass production whereas others were unaffected. Additionally, decreased flower and seed production was observed in all moderate overexpression plants.

The overexpression of miR156 in switchgrass results in delayed and decreased flowering. Several phenotypes observed in some of the low and moderate overexpression lines are desirable, including significantly delayed flowering. To optimize this system, miR156 expression levels may be fine-tuned through promoter selection that yields expression levels high enough to delay flowering without a negative impact on plant fitness. Delayed or decreased flowering could also be achieved by downregulating the *SPL* gene (s) that are targets of miR156, including *SPL 2/10* and *SPL 3/4/5* [86]. Temporal downregulation of these genes individually or in combination prior to floral transition could be achieved through RNA interference (RNAi) or CRISPR interference (CRISPRi) and result in delayed flowering without negatively affecting plant biomass production. Additionally, expression of other miRNAs known to regulate floral development can be altered to confer delayed flowering [87]. For example, miR172 expression is negatively correlated with miR156 expression, and overexpression of miR172 has been observed to accelerate flowering time in Arabidopsis [88]. Downregulation of miR172 could also confer similar phenotypes to those of miR156 overexpression and be useful for bioconfinement purposes.

## 5. Perspectives

GE crops that have been approved for commercial release are considered low risk of transgene flow and generally safe [17]. It is likely that regulatory bodies will require new GE crops with a high risk of gene flow or those that contain novel transgenic traits be put through lengthy and costly biosafety assessments before nonregulated status is approved. These crops would benefit from the inclusion of a bioconfinement system that significantly limits or eliminates transgene flow. In this review, we have highlighted the most prospective bioconfinement designs and many of these approaches have been tested in several plant species. Several designs have shown promise with nearly 100% bioconfinement efficacy. Should a system be adopted, the design must meet several key criteria. First, the system should be highly reliable, consistently reaching near 100% efficiency to reduce transgene exposure to unintended hosts. In addition, system components should not pose biosafety concerns regarding ecological risks, human health, or non-target organisms that may be exposed such as pollen-feeding insects. Lastly, it is vital that the bioconfinement system have no negative impact on crop yield, whether that is vegetative biomass, seed, or fruit yield. Many of the strategies reviewed here meet most of these criteria yet further improvements are needed for more robust and targeted gene expression. Indeed, the future is bright for this area of research and we expect advances in synthetic biology to be leveraged so new tools, such as inducible synthetic promoters, can be fine-tuned for robust gene expression in target tissue as well as improved efficiency of recombinases and CRISPR/Cas9-based methods. Although no technology is without risks, we are optimistic that several bioconfinement systems will soon be adopted that reduce biosafety concerns of stakeholders, regulators, and the public.

## Data Availability

Data sharing not applicable.
